# Vitamin D Endocrine System and COVID‐19

**DOI:** 10.1002/jbm4.10576

**Published:** 2021-11-17

**Authors:** Roger Bouillon, José Manuel Quesada‐Gomez

**Affiliations:** ^1^ Laboratory of Clinical and Experimental Endocrinology, Department of Chronic Diseases, Metabolism and Ageing KU Leuven Leuven Belgium; ^2^ Instituto Maimónides de Investigación Biomédica de Córdoba (IMIBIC), Hospital Universitario Reina Sofía Universidad de Córdoba, Fundación Progreso y Salud Córdoba Spain; ^3^ CIBER de Fragilidad y Envejecimiento Saludable (CIBERFES) Madrid Spain

**Keywords:** VITAMIN D, CALCIFEDIOL, COVID‐19, MORTALITY, ACUTE RESPIRATORY DISTRESS SYNDROME, INTENSIVE CARE TREATMENT

## Abstract

Preclinical data strongly suggest that the vitamin D endocrine system (VDES) may have extraskeletal effects. Cells of the immune and cardiovascular systems and lungs can express the vitamin D receptor, and overall these cells respond in a coherent fashion when exposed to 1,25‐dihydroxyvitamin D, the main metabolite of the VDES. Supplementation of vitamin D‐deficient subjects may decrease the risk of upper respiratory infections. The VDES also has broad anti‐inflammatory and anti‐thrombotic effects, and other mechanisms argue for a potential beneficial effect of a good vitamin D status on acute respiratory distress syndrome, a major complication of this SARS‐2/COVID‐19 infection. Activation of the VDES may thus have beneficial effects on the severity of COVID‐19. Meta‐analysis of observational data show that a better vitamin D status decreased the requirement of intensive care treatment or decreased mortality. A pilot study in Cordoba indicated that admission to intensive care was drastically reduced by administration of a high dose of calcifediol early after hospital admission for COVID‐19. A large observational study in Barcelona confirmed that such therapy significantly decreased the odds ratio (OR) of mortality (OR = 0.52). This was also the conclusion of a retrospective study in five hospitals of Southern Spain. A retrospective study on all Andalusian patients hospitalized because of COVID‐19, based on real‐world data from the health care system, concluded that prescription of calcifediol (hazard ratio [HR] = 0.67) or vitamin D (HR = 0.75), 15 days before hospital admission decreased mortality within the first month. In conclusion, a good vitamin D status may have beneficial effects on the course of COVID‐19. This needs to be confirmed by large, randomized trials, but in the meantime, we recommend (rapid) correction of 25 hydroxyvitamin D (25OHD) deficiency in subjects exposed to this coronavirus. © 2021 The Authors. *JBMR Plus* published by Wiley Periodicals LLC on behalf of American Society for Bone and Mineral Research.

## Introduction

Vitamin D, the threshold nutrient of the vitamin D endocrine system (VDES), is the precursor of 25 hydroxyvitamin D (25OHD), a prohormone and substrate for the synthesis of 1,25‐dihydroxyvitamin D (1,25(OH)2D), which binds with high affinity to its receptor (VDR), a member of the nuclear transcription factors. Liganded VDR regulates a very large number of genes,^(^ ^1^, ^2^ ^)^ mostly in coherent clusters. The major action of the VDR endocrine system (VDES/VDR) focuses on the intestine, where it stimulates active calcium absorption and thereby allows a normal bone development (and preventing rickets) or turnover (thereby slowing down bone loss and reducing the risk of osteoporosis). Most nucleated cells express VDR. Many cells also express CYP27B1, the enzyme responsible for the conversion of 25OHD into 1,25(OH)2D and are thus able to produce this hormone in an auto or paracrine fashion. Finally, most cells respond to VDR activation by changes in gene or protein expression or cell differentiation and function.^(^ ^1^, ^2^ ^)^

The immune cells are a well‐known target of the VDES, and 1,25(OH)2D regulates many crucial cytokines and metabolic signaling pathways of the innate and adaptive immune system.^(^ ^3^ ^)^ It is increasingly recognized that localized synthesis of 1,25(OH)2D in alveolar macrophages, dendritic cells, lymphocytes,^(4^ ^)^ as well as in airway epithelia, alveolar, and endothelial lung cells might be responsible, in an autocrine or paracrine fashion, for many of the immune effects of VDES. This includes the stimulation of proliferation, differentiation of alveolar cells, and the expression of some essential lung genes (including surfactant protein).^(^ ^5^ ^)^ The VDES also participates in the functional regulation of the cardiovascular system^(^ ^6^ ^)^ and is involved by several pathways in coagulation mechanisms.^(^ ^7^ ^)^

Therefore, the lung epithelium, immune, and cardiovascular systems that play a critical role in coronavirus disease 2019 (COVID‐19) infections^(^ ^8^ ^)^ are targets of vitamin D. Mild to severe vitamin D deficiency is highly prevalent around the world^(^ ^9^ ^)^ and has been associated with a large number of human diseases, including immune disorders and lung and cardiovascular diseases,^1^, ^10^ ^)^ and associated with idiopathic lower‐extremity deep vein thrombosis.^(^ ^11^ ^)^ It is therefore no surprise that already early during the COVID‐19 pandemic, a possible link between a poor vitamin D status and COVID‐19 infection or severity of its complication has been suspected. This resulted in more than 700 PubMed publications dealing with “vitamin D” and “COVID‐19” since the start of this pandemic.

This review aims to summarize, first, the available data regarding possible mechanisms whereby the VDES might interfere or protect against COVID‐19 or its complications. Second, we will review the clinical data (observational and intervention studies) linking VDES with COVID‐19.

## Potential Mechanisms Linking Vitamin D and COVID‐19 Infection

### Vitamin D and the immune system

**Fig. 1:**
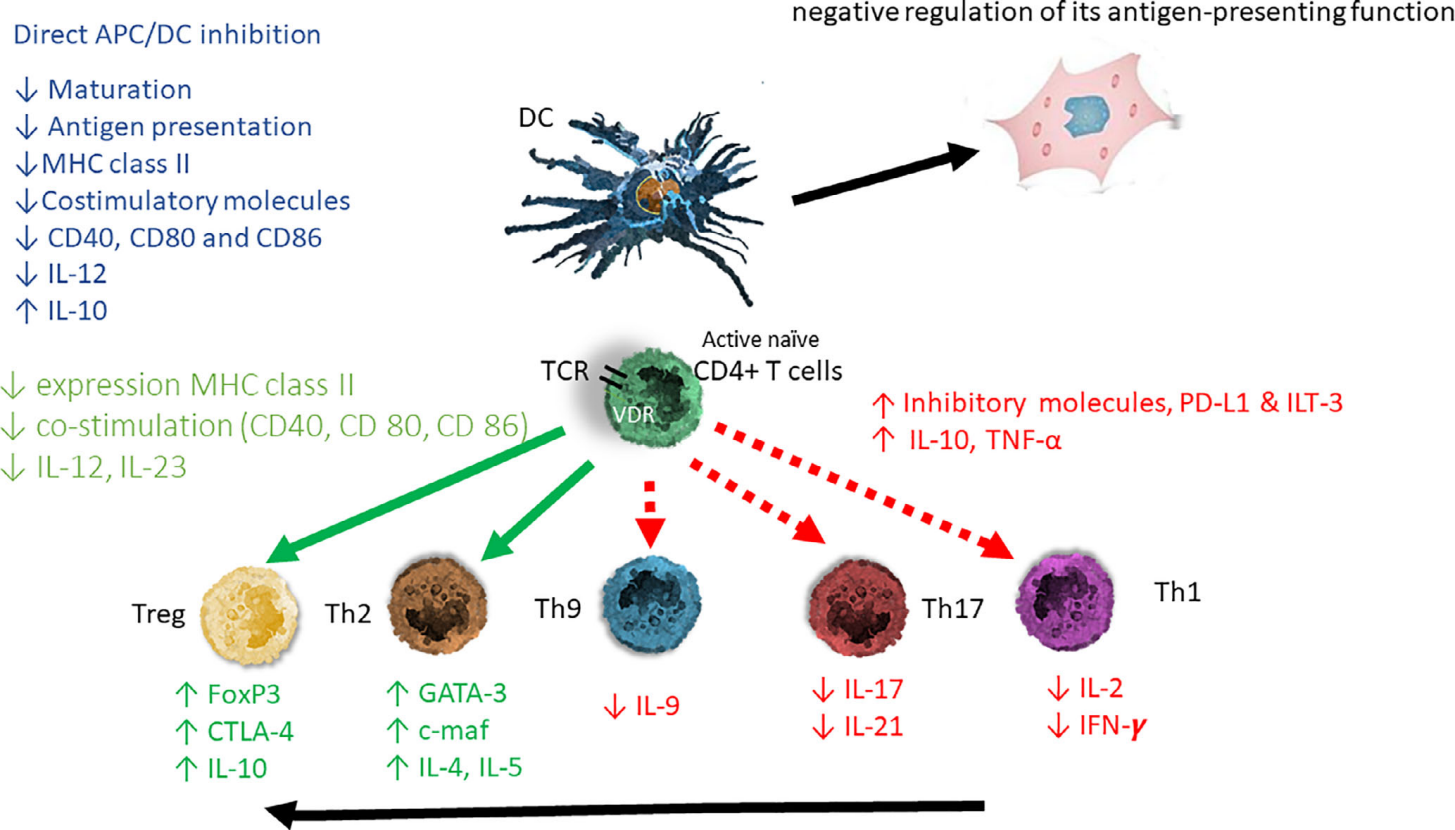
Schematic presentation of the immune modulating activity of the vitamin D endocrine system. 1,25(OH)2D modulates dendritic cells (DC) toward a less mature and more tolerogenic phenotype with changes in both morphology (more adherent spindle cells), cytokine production, and surface markers with a negative regulation of their antigen‐presenting function. 1,25(OH)2D exerts its effect through direct binding to the vitamin D receptor (VDR) of the antigen‐presenting cell (APC) and to activated T lymphocytes. T‐cell receptor (TCR) signaling induces upregulation in the vitamin D receptor (VDR). 1,25(OH)2D exerts on DC a direct inhibition of APC and a negative regulation of its antigen presentation function. The DC‐derived cytokines will alter the T‐helper (Th) lymphocyte balance from a Th1 and Th17 predominance toward a Th2 phenotype. The direct effect of 1,25(OH)2D on the T lymphocytes creates a change toward a more tolerogenic state with an induction of Thelper‐2 (Th2) lymphocytes and regulatory T lymphocytes (Tregs); depicted in green arrow, together with a downregulation of the pro‐inflammatory Thelper‐1 (Th1) lymphocytes, Thelper‐17 (Th17) lymphocytes, and Thelper‐9 (Th9) lymphocytes (depicted in red arrow). APC = antigen‐presenting cell; DC = dendritic cell; naïve T cells MHC = membrane histocompatibility complex; cluster of differentiation (CD) 80 = CD86 (co‐stimulatory molecules), and CD54 (adhesion molecule); PD‐L1 = programmed death‐ligand 1; ILT‐3 = immunoglobulin‐like transcript, T lymphocytes; TH1 = T helper 1; TH2 = T helper 2; TH17 = T helper 17; Treg = regulatory T cell; IL = interleukin; TNF‐α = tumor necrosis factor‐α; FoxP3 = forkhead box P3 (master gene controlling the development and function of regulatory cells); CTLA‐4 = cytotoxic T lymphocyte‐associated Ag‐4).

All cells of the immune system express VDR at a certain time during their lifetime, including thymocytes, monocytes and macrophages, antigen‐presenting cells, T and B cells, neutrophils, and mast cells. Multiple and rapidly expanding studies are demonstrating that VDES/VDR signaling is active in both the innate and adaptive arms of the immune system^(^ ^12^ ^)^ (Fig. 1). {FIG1}

#### Monocytes and macrophages

Monocytes and macrophages constitutively express VDR. Exposure to 1,25(OH)2D enhances macrophage differentiation from monocytes, but as monocytes differentiate toward macrophages, there is a decrease in the expression levels of the VDR.^(^ ^13^ ^)^ Serum 25OHD not bound to vitamin D‐binding protein (DBP) allows intracellular access of 25OHD for conversion to 1,25(OH)2D, which then binds to VDR^(^ ^14^ ^)^ with increased expression of VDR, and CYP27B1, which raised intracellular production of 1,25(OH)2D from its substratum 25OHD.^(^ ^4^ ^)^ 1,25(OH)2D has a direct antimicrobial role in monocytes and macrophages by induction of cathelicidin antimicrobial peptide (CAMP),^(^ ^15^ ^)^ with an increase of hCAP18 and LL‐37, and by targeting defensing β2 (HBD2 /DEFB4). This results in increased clearance of intracellular bacteria (such as mycobacterium tuberculosis) and defense against viral infections.^(^ ^16^ ^)^ Cathelicidin not only has a negative role on bacterial and viral proliferation but also enhances local inflammation and leucocyte migration as to eliminate the invasion by pathogens.^(^ ^17^ ^)^

The 1,25(OH)2D‐VDR complex in addition to induction of antimicrobial peptides strongly induces CD14, TLR4 co‐receptor,^(^ ^18^ ^)^ and IL‐1β and IL‐8 cytokine signaling,^(19^ ^)^ and for some responses (eg, DEFB4 induction), accessory immune signaling (MDP binding to NOD2 requires the combination of IL‐1β with the 1,25(OH)2D‐VDR complex through nuclear factor κB (NF‐κB).^(^ ^20^ ^)^ However, the 1,25(OH)2D‐VDR complex suppresses the expression of hepcidin antimicrobial peptide (HAMP), which regulates the intracellular concentration of iron, via ferroportin, necessary for bacterial growth.^(^ ^21^ ^)^

1,25(OH)2D also has an antioxidative effect on monocytes by upregulation of glutathione reductase (GR) and glutamate‐cysteine ligase (GCL), resulting in reduced production of oxygen radicals.^(^ ^22^ ^)^

In addition, these cells can start to express the 1α‐hydroxylase enzyme, CYP27B1, after exposure to immune stimuli (ie, signal transducer and activator of transcription‐1α [STAT‐1α], interferon‐γ [IFN‐γ], lipopolysaccharide [LPS], and toll‐like receptor [TLR]).^(^ ^23^ ^)^ The locally produced 1,25(OH)2D action then, typically after a few days, results in an anti‐inflammatory activity on macrophages as it increases interleukin (IL)‐10 and decreases inflammatory stimuli (ie, IL‐1β, IL‐6, tumor necrosis factor‐α [TNF‐α], receptor activator of nuclear factor kappa‐B ligand [RANKL], and cyclo‐oxygenase‐2 (COX‐2).^(^ ^24^ ^)^

#### Antigen‐presenting cells

Antigen‐presenting or dendritic cells (DCs) play a crucial role in the activation (or not) of most T cells. 1,25(OH)2D directs DCs toward a less mature and more tolerogenic phenotype as shown by a different morphology (spindle‐shaped cells) and by altered cytokine production and changes in the expression of surface markers.^(^ ^13^ ^)^ At the cell surface, major histocompatibility complex (MHC) II, cluster of differentiation (CD) 80, CD86 (co‐stimulatory molecules), and CD54 (adhesion molecule) are less expressed, whereas CCR5 (chemokine receptor), DEC205 (antigen‐uptake receptor), F4/80 (macrophage marker), and CD40^(^ ^25^ ^)^ show increased presence. 1,25(OH)2D decreases the expression and secretion of several cytokines (IL‐6 and IL‐12) together with an increase in IL‐10, a tolerogenic cytokine. Finally, 1,25(OH)2D stimulates the induction of regulatory T cells (Tregs) mediated by induction of programmed death‐ligand 1 (PD‐L1)^(^ ^26^, ^27^ ^)^ and TNFα (Fig. 1). By all these actions, 1,25(OH)2D decreases the presentation of antigens.

#### Neutrophils

Neutrophils are short‐lived cells, represent the largest number of circulating immune cells, and accumulate in areas targeted by pathogens aiming to destroy invading microorganisms by oxidative burst and phagocytosis. They express VDR at a level comparable to monocytes and are reactive to 1,25(OH)2D^(^ ^28^ ^)^ and thereby tend to decrease the mobility of neutrophils and taper down the inflammatory reaction to pathogens and exhibit 1,25(OH)2D‐driven expression of CD14 and CAMP,^(^ ^29^ ^)^ although IL‐1β was slightly inhibited. Neutrophils are the main source of cathelicidin/LL37 in blood, due to their abundance and the presence of neutrophil granules that store most of the LL37 released at sites of infection. Unlike monocytes/macrophages and dendritic cells, however, neutrophils do not appear to express CYP27B1, indicating that they are more likely systemic responders to the hormonal 1,25(OH)2D. Neutrophils can also form neutrophil extracellular traps (NETs), web‐like extracellular fibers composed of DNA, histones, enzymes, and proteins of pathogens, able to immobilize and expel these invaders. The role of VDES activation in neutrophils has not been extensively studied. One study reported that 1,25(OH)2D (in vitro) increased NET formation in neutrophils from normal subjects.^(^ ^30^ ^)^ However, 1,25(OH)2D could reduce endothelial damage by decreasing NETosis activity.^(^ ^31^ ^)^ Overall, these findings suggest a role for VDES/VDR in dampening neutrophil‐driven inflammatory responses, while still boosting pathogen killing by the cells T lymphocytes.

#### T cells

In naïve T cells, the VDR is expressed at very low levels or is even undetectable, but after activation, in response to T‐cell receptor (TCR) signaling mediated by the p38 MAPK pathway, VDR expression is significantly increased.^(^ ^32^ ^)^ VDR is stabilized from degradation by 1,25(OH)2D binding. Activated T cells express CYP27B1, which mediates the intracellular conversion of 25OHD to 1,25(OH)2D, which stimulates intracrine activation of the VDR.^(^ ^32^ ^)^ The direct effect of 1,25(OH)2D will vary in intensity depending on the degree of T‐cell activation, since the intensity of activation modulates the VDR concentration.

Most of the effects of 1,25(OH)2D on T lymphocytes are due to its indirect effects on antigen‐presenting cells, thereby, as described above, downregulating the activation of Th cells by a variety of mechanisms (cell–cell contacts and cytokines). This results in decreased antigen presentation, decreased IL‐12 and IL‐23 production, and enhanced IL‐10 and macrophage inflammatory protein MIP‐3a^(^ ^33^ ^)^ (Fig. 1). 1,25(OH)2D produced by monocytes/macrophages dramatically changes the immune state from a pro‐inflammatory to a tolerogenic state. 1,25(OH)2D suppresses T‐lymphocyte proliferation and modulates cytokine production and differentiation with diverse effects on different T‐lymphocyte subsets.^(^ ^34^ ^)^

Overall, the balance of the adaptive immune system switches from lymphocytes TH1, TH9, and TH17 to TH2 immune profile by suppressing the expression of TH1 (IL‐2, IFN‐γ, TNF‐α), TH9 (IL‐9), and TH17 (IL‐17, IL‐21) cytokines, while inducing the expression of TH2 cytokines (IL‐4, IL‐5, IL‐9, IL‐13). Some direct effects of 1,25(OH)2D on T cells enforce its indirect action, such as direct inhibition of IL‐2 and gamma interferon and probably an increased production of IL10; CCR6 on Th17 cells is also suppressed, preventing them from directing to tissues^(^ ^4^, ^12^ ^)^ (Fig. 1). 1,25(OH)2D also promotes differentiation of regulatory T cells (Treg) both directly and indirectly through its interaction with antigen‐presenting cells, resulting in a suppression of proinflammatory state.^(^ ^35^ ^)^

Because 1,25(OH)2D is only induced in the immune system with a long lag time of several days after contact with pathogens, it will not impede the early immune defense against pathogens but taper down excessive immune reactions to pathogens. Such excessive immune reaction is potentially deleterious as shown by excessive systemic and local inflammation.

#### B lymphocytes

Inactive B lymphocytes do not have VDR, but when activated, B lymphocytes express both VDR and CYP27B1.^(^ ^36^ ^)^ 1,25(OH)2D activation of VDR seems to stimulate B‐lymphocyte apoptosis^(^ ^37^ ^)^ and impedes the generation of plasma cells (by modulation of CD40 and thus NF‐κB), modulating antibody production by plasma cells.^(^ ^38^ ^)^ 1,25(OH)2D additionally upregulates IL‐10 production by B lymphocytes, providing an enhanced immunoregulatory effect.^(^ ^39^ ^)^ Furthermore, 1,25(OH)2D reduces T‐cell activation by B cells (by downregulating CD86 expression and upregulating CD74).^(^ ^24^ ^)^ Overall, all actions of 1,25(OH)2D on the immune system is a combination of stimulation of the innate immune defense and with some delay it tapers down the activation of the acquired immune system, activation of tolerogenic cells. It also decreases inflammation by mediation of a wide variety of cytokines.

### Vitamin D and the lung

VDR is already expressed in alveolar type 2 cells during fetal life^(^ ^40^ ^)^ during the last quarter of gestation and CYP27B1 and CYP24A1 just before birth. VDR is thus expressed in the lung epithelium before it is expressed in the intestine. Later on in life, human bronchial epithelial cells express VDR, CYP27B1, CYP24A1, and cathelicidin as demonstrated in non‐used human lung transplant tissue.^(^ ^41^ ^)^ VDR is especially expressed in apical epithelial cells but not in endothelial cells. Lung fibroblasts and smooth muscle cells as well as alveolar macrophages also express VDR. The latter cells also express CYP27B1 after stimulation by toll‐like receptors, interferon‐gamma, or LPS.^(^ ^5^ ^)^ These epithelial barrier cells also express cathelicidin (as described in “Monocytes and macrophages”) after exposure to 1,25(OH)2D with beneficial defense against foreign pathogens.

The appearance of VDR during gestation coincided with the start of type 2 pneumocyte differentiation and secretion of surfactant factor. Moreover, in cultures of rat fetal lung explants or freshly isolated cells, exogenous 1,25 (OH)2D3 accelerated the maturation of the type 2 pneumocytes through reduction in their glycogen content and increase in their surfactant synthesis and secretion.^(^ ^42^ ^)^ Whether these observations have functional implications has been evaluated in animal models. Vitamin D‐deficient rodents show abnormal airway and alveolar development, with altered lung function and airway hyperreactivity in their offspring.^(^ ^43^ ^)^ Whole lung transcriptome alterations in progenies from maternal vitamin D‐deficient animals indicated transcript level changes related to abnormal lung growth/development and activation of innate immunity and stimulation of inflammation.^(^ ^44^ ^)^ In humans, maternal vitamin D deficiency has been associated with postnatal impairment in lung function in childhood and with increased risk for developing wheezing early in life.^(^ ^45^ ^)^ Intervention studies with high‐dose vitamin D supplementation during pregnancy, however, decreased (just significantly) wheezing or asthma‐like symptoms in early childhood but no longer in older offspring.^(^ ^46^ ^)^ In full‐term infants, low levels of 25OHD in cord blood were shown to be associated with poor lung function performance assessed using infant lung function testing and increased respiratory infection in infancy. These observations suggest an important role of the vitamin D endocrine system in fetal and early life lung maturation and function with possible longer‐term consequences.

**Fig. 2:**
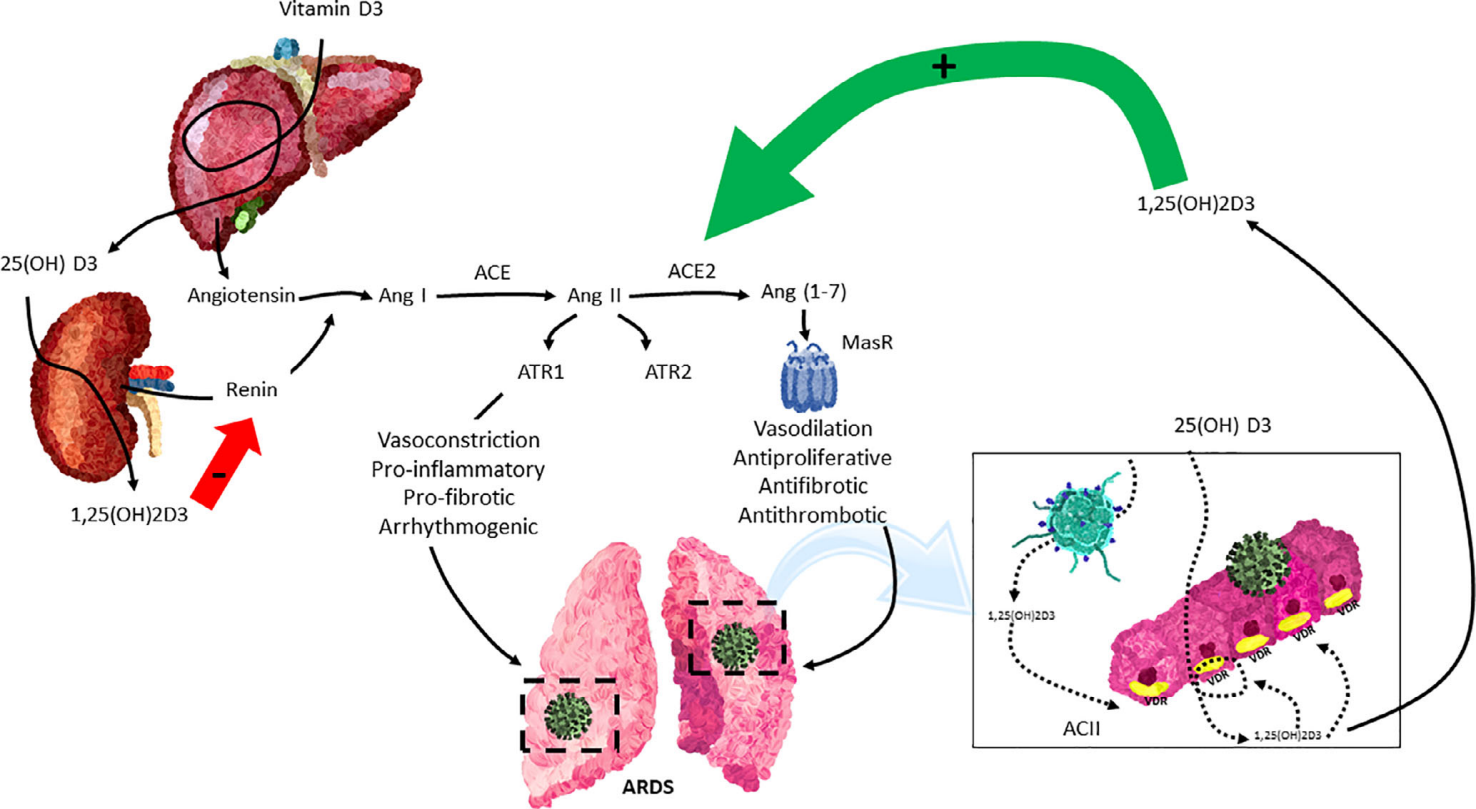
The vitamin D endocrine system tapers down the acute respiratory distress syndrome (ARDS). The vitamin D receptor (VDR) and enzymes of the vitamin D endocrine system are expressed in the cuboidal alveolar type 2 cells (ACII) and monocyte/macrophages/granulocytes and activated lymphocytes. The availability of 25OHD3 (calcidiol) is critical for synthesizing 1,25(OH)2D (calcitriol), which through endocrine, auto/paracrine action on VDR: (1) decreases the intensity of cytokine and chemokine storm, (2) modulates neutrophil activity, (3) maintains the integrity of the pulmonary epithelial barrier, (4) stimulates epithelial repair, and (5) decreases directly and indirectly the risk of hypercoagulability and pulmonary or systemic thrombosis. SARS‐CoV‐2 = severe acute respiratory syndrome coronavirus 2; IFN‐α, IFN‐γ = interferon gamma α and γ; IL‐1β, IL‐6, IL‐12, IL‐18, IL‐33 = interleukin‐1β, 6, 12, 18, 33; TNF‐α = tumor necrosis factor‐α; TGFβ = transforming growth factor α and β; CCL2, CCL3, CCL5 Chemokine = C‐C motif ligand 2, 3, 5; CXCL8, CXCL9, CXCL10 = C‐X‐C (motif chemokine ligand 8, 9, 10).

The VDR endocrine system may continue to have an influence on adult lungs. In vitro studies suggested an antiviral activity of high concentrations of 1,25(OH)2D as demonstrated by decreased proliferation of rhinoviruses in cultures of human primary bronchial epithelial cells. In addition, VDR activation decreased viral‐induced inflammatory responses even in the absence of effects on some specific viruses.^(^ ^5^ ^)^ These data at least suggest that vitamin metabolites may be beneficial in clearing pathogens (bacterial or viral) and decrease the inflammatory response of the host against these invaders. Other studies suggest that 1,25(OH)2D may decrease disease‐induced increased remodeling and fibrosis of lung tissues, most likely linked to the well‐known antiproliferative and anti‐inflammatory effects of the vitamin D endocrine system (Fig. 2). {FIG2}

VDES has been demonstrated to regulate the components of tight junctions and maintain the integrity of epithelial barriers in multiple organs.^(^ ^47^, ^48^, ^49^ ^)^ In the gut, VDES is known to strengthen the epithelial barrier structure,^(49^ ^)^ and it seems that it has the same effects in the lung. As in the intestine, the expression of several claudins is impaired in the absence of VDES action. The same has been observed in the lung epithelium of VDR‐deficient mice, resulting in reduced tight junctions and compromised pulmonary barrier integrity.^(^ ^50^ ^)^ In line with these data, several studies suggest that VDES may improve lung barrier repair or impair tissue damage. In a neonatal mouse model, vitamin D reduced the tissue damage induced by exposure to hyperoxia.^(^ ^51^ ^)^ In an LPS‐induced lung injury model, 1,25(OH)2D attenuated lung injury by inhibition of apoptosis and epithelial‐mesenchymal transition.^(^ ^52^ ^)^

Many clinical studies focused on the relation between vitamin D status and upper respiratory infection. Many observational studies found a higher risk of infections in subjects with a poor vitamin D status, including bacterial infections such as tuberculosis and viral infections such as HIV or upper respiratory infections. Causality is less evident for tuberculosis infections, but several large meta‐analyses showed a significant reduction in the risks of upper respiratory infections by daily or weekly vitamin D supplementation, especially in subjects with a poor baseline vitamin D status.^(^ ^53^, ^54^ ^)^

### COVID‐19 and vitamin D

#### Possible mechanism of interaction

Several recent reviews have tried to identify several mechanisms of interaction between SARS‐CoV‐2 infection and the VDES (Fig. 2).^(^ ^55^, ^56^, ^57^ ^)^ First, in line with the stimulatory effect of VDR activation on the native immune system, it could be that monocytes/macrophages and their defensins, stimulated by 1,25(OH)2D, would decrease the risk viral spread into the host.^(^ ^58^ ^)^ This would be in line with the beneficial effects of vitamin D supplementation of subjects with rather poor vitamin D status on the risk of upper respiratory infections. The observational data, described below, would be in line with this hypothesis. Unfortunately, the in vitro or preclinical data on the protection provided by 1,25(OH)2D on viral infections is limited, as described above, as it inhibits some but not all viral infections and there are no reliable data on 1,25(OH)2D's effect on in vitro infections of human cells by COVID‐19. Apart from its action on defensins, 25OHD and 1,25(OH)2D may also stimulate autophagy by a variety of targets;^(^ ^59^, ^60^ ^)^ thus, autophagy may be sensitive to changes in 25OHD serum level mechanisms including downregulation mTOR (an anti‐apoptotic agent)^(^ ^61^ ^)^ and stimulation of pro‐apoptotic proteins (such as Beclin 1 and PI3KC).^(^ ^62^ ^)^ This then results in elimination of infected cells and its pathogens. As described below, however, clinical observations and trials have so far not convincingly shown that a good vitamin D status is playing a substantial role in the primary prevention of COVID‐19 infections.

Severe COVID‐19 most often involves respiratory manifestations, although other organs and systems are also affected, and acute illness is often followed by prolonged complications. Such complex manifestations suggest that SARS‐CoV‐2 dysregulates the host response, triggering broad‐spectrum immuno‐inflammatory, thrombotic, and parenchymal disorders.

**Fig. 3:**
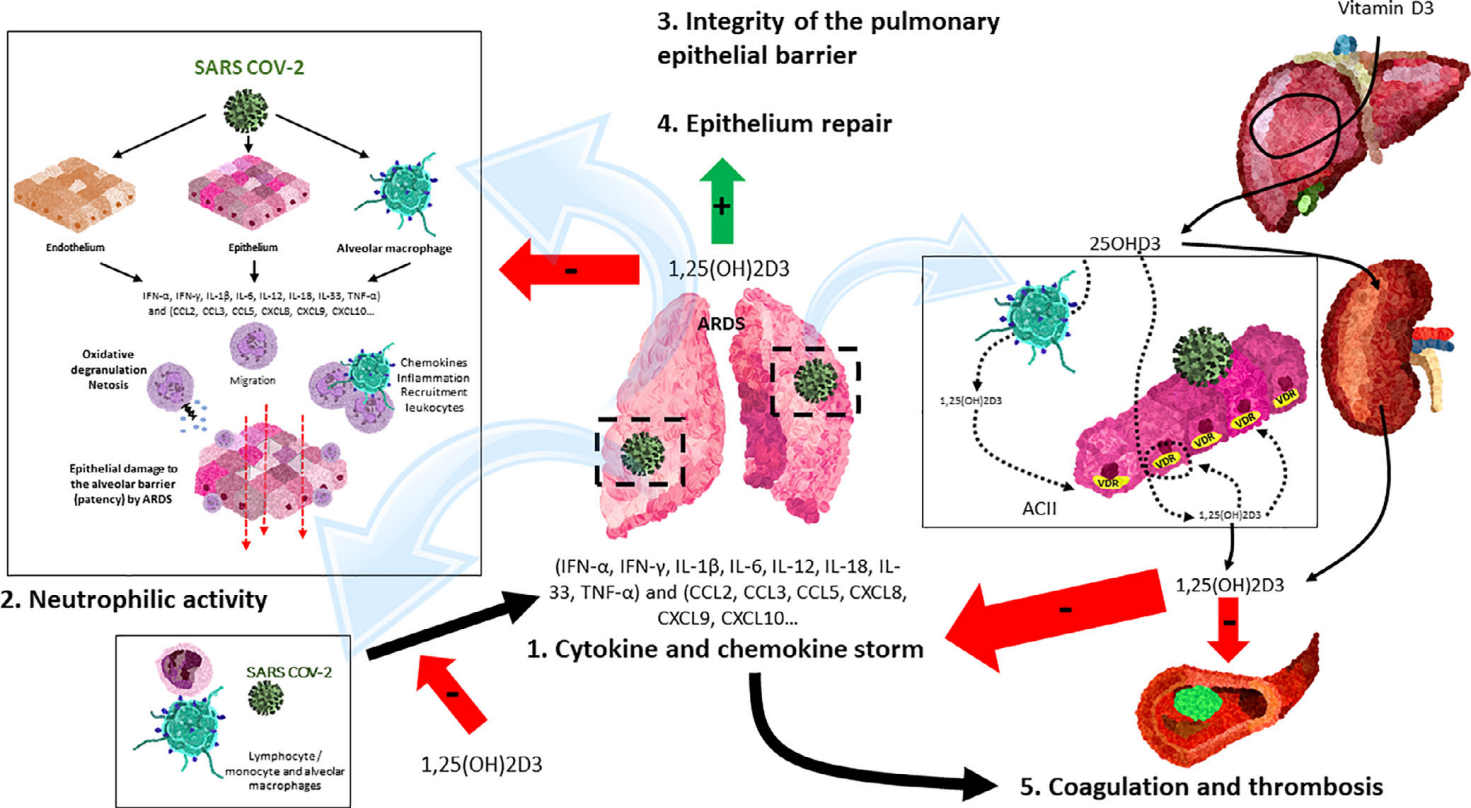
Vitamin D endocrine system and the renin‐angiotensin system. Local or systemic inflammatory reactions may activate the renin‐angiotensin system (RAS) and ACE, thereby generating angiotensin II, which via its receptor (ATR) is able to induce lung damage. During SARS‐CoV‐2 invasion, ACE2 is downregulated in type II alveolar epithelial cells, thereby decreasing the conversion of Ang II to Ang‐(1–7). This prevents the protective action of the Ang‐(1–7) acting on its receptor (Mas R) and all aspects of ARDS. 1,25(OH)2D/VDR is a powerful negative regulator of the RAS, inhibiting renin and the ACE/Ang II/AT1R cascade and inducing ACE2/Ang‐(1–7) axis activity. ACII = cuboidal alveolar type II cells; SARS‐CoV‐2 = severe acute respiratory syndrome coronavirus 2; Ang I = angiotensin I; Ang II = angiotensin II; Ang‐(1–7) = angiotensin 1–7; MasR = G protein‐coupled Mas receptor; AT1R and AT2R = angiotensin II receptor 1 and 2.

Much more attention has been given to the possible effects of vitamin D status on the severity of COVID‐19 infection, especially on the need for hospitalization, the need for intensive care (and respiratory assistance), or mortality. Although COVID‐19 may cause serious problems in different tissues, especially the ones that express ACE‐2, including heart problems, the major tissue affected is the lung. Indeed, some patients with severe acute respiratory syndrome coronavirus 2 (SARS‐CoV‐2) infections develop severe pneumonitis with pulmonary inflammation, thick mucus secretions in the airways, elevated local and systemic levels of proinflammatory cytokines, extensive lung damage, and microthrombosis. This is also known as acute respiratory distress syndrome (ARDS) and develop in 10% to 25% of patients hospitalized because of COVID‐19. Such patients require oxygen therapy and may require assisted ventilation or similar invasive techniques and ultimately many of such patients die from this complication and multiorgan failure. ARDS due to COVID‐19 is very similar to other situations regardless of the initial trigger and may rapidly aggravate after an initial more benign course of the infection. Early recognition of ARDS is critical as treatment in later stages is problematic. The mechanisms underlying the pathogenesis of ARDS have been extensively studied, as they are not specific for COVID‐19. Several factors contribute to this severe complication, including:

1. Cytokine and chemokine storm (Fig. 2). The normal host response to viral infection results in upregulation of interferon transcription and activation of transcription factors, such as nuclear factor kappa B (NF‐κB), which stimulates the release of various cytokines to mobilize monocytes, macrophages, lymphocytes, and neutrophils to respond to viral infection.^(^ ^63^ ^)^ We now know that coronaviruses have evolved several mechanisms to evade detection and targeting of the host response, shortly after infection.^(^ ^64^ ^)^ Cytokine and chemokine production in COVID‐19 occurs via two pathways: by direct viral recognition by immune cells through pattern recognition receptors, prominently virus‐specific toll‐like receptors (TLR3, TLR7, TLR8, and TLR9), and indirectly through the mediation of damage‐associated molecular patterns (DAMPS) released from epithelial cells damaged by SARS‐CoV‐2.^(^ ^65^ ^)^ Patients with severe COVID‐19 have restricted or delayed responses to type 1 and type 2 interferon,^(^ ^66^ ^)^ and therefore the inflammatory reaction is necessary to combat the invasion by pathogens, but excessive inflammation with massive release of cytokines (including several interferons and interleukins) and chemokines (including several CCLs and CXCLs) may not be necessary and can become deleterious by itself. In fact, severe COVID‐19 presents a cytokine storm with elevated plasma levels of chemokine ligand 2 (CCL2), IFNγ, IFNγ‐inducible protein 10, G‐CSF, C‐C motif chemokine ligand 3 (CCL3), IL‐1β, IL‐2, IL‐6, IL‐7, IL‐8, IL‐10, IL‐17, and TNF‐α.^(^ ^67^ ^)^ However, surprisingly, systemic inflammation during COVID‐19 is usually less intense than in other inflammatory syndromes.^(^ ^68^, ^69^ ^)^ These interleukins and chemokines recruit inflammatory cells (neutrophils, T lymphocytes, and NK cells) to the sites of inflammation and may aggravate the pneumonitis and its consequences.^(^ ^55^, ^56^ ^)^ On the other hand, intense oxidative stress induced by high cytokine concentrations, together with reduced concentrations of interferon α and interferon β (IFN‐α, IFN‐β), influences the expression of COVID‐19 severity.^(^ ^67^ ^)^ Activation of the VDR system may taper down inflammation and thus, like glucocorticoids, reduce the excessive inflammation characteristic for ARDS,^(^ ^70^ ^)^ but unlike corticosteroids, the activation of the VDES does not inactivate innate immunity but stimulates it^(^ ^4^, ^12^, ^71^, ^72^ ^)^ (Fig. 2).
2. Activation of the renin angiotensin system (RAS). Local or systemic inflammatory reactions may activate the RAS, whereby angiotensin II generated by ACE is able to induce lung damage (Fig. 3). {FIG3} In contrast, ACE2 transforms angiotensin II into smaller peptides with lung‐protective effects. The alteration of the balance between the levels of the enzymes ACE1 and ACE2 alters the ratio of Ang II: Ang‐(1‐7), whereby increased angiotensin II is key to the development of ALI and ARDS in animal models and humans.^(^ ^73^, ^74^ ^)^ In addition, SARS‐CoV‐2 uses human ACE2 receptor for viral entry and cell tropism for infectivity.^(75^ ^)^ A viral spike (S) glycoprotein mediates viral entry by binding to ACE2 on the epithelial cell surface, a process supported by transmembrane serine protease 2 (TMPRSS2). Most alveolar epithelial cells express this receptor and thus facilitate the cellular entry of this virus. SARS‐CoV‐2 may decrease the cell surface expression of ACE2 and thereby decrease a lung‐protective mechanism, resulting in greater inflammation, edema, and more severe ARDS. VDR activation downregulates ACE1 (and its pro‐inflammatory consequences) but also upregulates ACE2 (Fig. 3).
  1,25(OH)2D is a potent negative regulator of the renin‐angiotensin system (RAS). Indeed, renin is increased in VDR null mice^(^ ^76^ ^)^ and mice deficient in 1α‐hydroxylase show increased intrarenal RAS activity that is reduced by 1,25(OH)2D administration.^(^ ^77^ ^)^ Chronic 25OHD deficiency appears to induce RAS activation.^(^ ^78^ ^)^ 1,25(OH)2D inhibits renin, ACE, and Ang II expression and induces ACE2 levels in LPS‐induced respiratory distress. Furthermore, dysregulation of local and circulating RAS, with increased ACE/Ang II expression levels and reduced ACE2/Ang‐(1‐7) expression levels, was reported to contribute to ischemia‐reperfusion‐induced acute lung injury (ALI) in mice.^(^ ^79^ ^)^ Therefore, vitamin D may attenuate LPS‐induced ALI, at least partially, by inducing ACE2/Ang‐(1‐7) axis activity and inhibiting renin and the ACE/Ang II/AT1R cascade (Fig. 2*B*).^(^ ^80^ ^)^ VDR activation is also able to inhibit the Skp2 protein,^81^, ^82^ ^)^ which plays a pivotal role in the mechanism of viral replication in COVID‐19. Indeed, SARS‐2 uses blockade of autophagy to accelerate its replication and infectivity.^(^ ^83^ ^)^ To achieve this, the virus induces Skp2, which, in turn, inactivates Beclin 1, an essential component of the autophagic process. 1,25(OH)2D also stimulates the production of Klotho, which is known to attenuate multiorgan aging and increase longevity, and also promotes autophagy through the maintenance of adequate cellular levels of Beclin.^(^ ^84^ ^)^
3. Neutrophils are an important part of the defense against viral pathogens but also of tissue damage. The release of neutrophil chemoattractant cytokines and chemokines and the resulting neutrophil recruitment are a global host response to SARS‐2 infection. There is a passage of mature neutrophils from the bone marrow to the circulation and from the circulation to the tissues, especially the lungs (and kidneys), which represent the main targets in COVID‐19. The occurrence of immature neutrophil subsets (preNeu and immature) in the blood and the increased neutrophil‐to‐lymphocyte ratio (NLR),^(^ ^85^ ^)^ a consistent clinical biomarker of inflammation,^(86^ ^)^ predict disease severity in the early phase of SARS‐CoV‐2 infection
  In COVID‐19, as in other viral infections, the main function of neutrophils is virus and debris clearance through phagocytosis, release of cytokines and defensins, degranulation, oxidative burst, and neutrophil extracellular traps (NETs).^(^ ^87^ ^)^ NETs can exert both proinflammatory and anti‐inflammatory effects, which depend on the course of the disease.
  Proinflammatory effects include induction of type 1 interferons (IFNs) and proinflammatory cytokines, induction of the NLRP3 inflammasome, promotion of adaptive immune responses, and endothelial damage with involvement in immunothrombosis.^(^ ^88^ ^)^
  In COVID‐19, massive infiltration of activated neutrophils into the lungs is accompanied by the formation of NETs as possible drivers of ARDS with immunothrombosis leading to occlusion of pulmonary vessels The lung alveoli can be filled with activated neutrophils and protein‐rich edema. In addition, the barrier function of the lung epithelium is damaged. In this state, the lungs cannot provide enough oxygen to the blood for the body's vital organs, all contributing to ARDS. NETS play a pivotal role in the widespread organ damage with involvement not only of lung tissue but also of the kidney and liver.^(^ ^89^ ^)^ This link between NETs and thrombosis may also be related to the activation of the complement system, suggesting that the thrombotic effects of NETs may be related to the systemic and deleterious effects of COVID‐19.^(^ ^90^ ^)^
4. Coagulation. Coagulopathy and endothelial damage are important conditions occurring in severe COVID‐19, with frequent reports of arterial and venous thromboembolism.^(^ ^91^ ^)^ Patients with severe COVID‐19 often present with signs of hypercoagulability, ie, very high circulating D‐dimer concentrations (3 to 40 times normal concentrations), increased fibrinogen, increased prothrombin time and activated partial thromboplastin time, and thrombocytopenia are commonly observed in critically ill COVID‐19 patients.^(^ ^92^ ^)^
  Possible key factors in hypercoagulability in COVID‐19 are direct virus‐induced endothelial damage and the resulting inflammation (mediated by cytokines, reactive oxygen species, and acute‐phase reactants and NETs). COVID‐19‐associated endotheliitis with diffuse microcirculatory injury in the lungs appears to be a central feature of the severe COVID‐19 phenotype.^(^ ^93^ ^)^
  Intensive inflammation and activation of RAS can alter the coagulation cascade. In combination with endothelial cell infection, this results in a more prothrombotic status as found in ARDS. Such increased coagulation cascade and the subsequent formation of intra‐alveolar or systemic fibrin clots and thrombotic complications are prominent findings in some patients with SARS‐CoV‐2 infections. The vitamin D endocrine system plays an important role as an anti‐inflammatory and anti‐thrombotic agent: 1,25(OH)2D (i) suppresses the inflammatory response of effector T cells by inhibiting the maturation and activity of dendritic cells in a VDR‐dependent manner; (ii) activates anti‐inflammatory IL‐10 production in B cells; (iii) downregulates TNF, IL‐6, NF‐κB, MCP‐1, and activates the antimicrobial peptide cathelicidin in macrophages; (iv) downregulates IFNγ, IL‐17, and IL‐21 in T cells; (v) upregulates the natural anticoagulants thrombomodulin (TM) and tissue factor pathway inhibitor (TFPI), and deactivates tissue factor (TF) and thereby reduces hypercoagulability.^(^ ^7^ ^)^
  The antithrombotic effects of VDES are well documented in preclinical studies. VDR knockout mice show increased platelet aggregation. Their gene expression of antithrombin (liver) and thrombomodulin (aorta, liver, and kidney) was downregulated, whereas tissue factor expression in liver and kidney was upregulated. Stimulation of VDR with 1,25(OH)2D or its agonists decreased thrombomodulin and thrombomodulin gene expression in monocytic cells previously stimulated by tumor necrosis factor (TNF), lipopolysaccharide (LPS), and oxidized LDL (ox‐LDL).^(^ ^94^ ^)^ VDR KO mice manifested exacerbated multiorgan thrombus formation after exogenous lipopolysaccharide injection with increased endothelial adhesion molecules, decreased NO production, and increased platelet aggregation.^(^ ^95^ ^)^ 1,25(OH)2D and paricalcitol significantly reduced TF expression and its procoagulant activity, induced by the proinflammatory cytokine TNF‐α in human aortic vascular smooth muscle cells (VSMC), in an NF‐κB‐dependent manner. This was accompanied by positive upregulation of the TF signaling mediator protease‐activated receptor 2 (PAR‐2).^(^ ^96^ ^)^
  The antithrombotic effects of VDES have been confirmed in several clinical studies, which correlated 25OHD deficiency with thrombosis. Observational data in humans revealed an association between low levels of 25OHD and the development of deep venous thromboembolic events (DVTE) in patients with ischemic stroke.^(^ ^97^ ^)^ On the other hand, a significant positive association was found between serum 25OHD levels (>20 ng/mL) and TF pathway inhibitor (TFPI), a dual inhibitor of coagulation by binding to both the TF/Factor VIIa and Factor Xa complex.^(^ ^98^ ^)^
  In the longer term, this also increases the risk of fibrosis and impaired lung function after recovery of the disease.
5. Lung fibrosis. Approximately half of patients with severe COVID‐19 develop ARDS, and a recent systematic review has shown that approximately 20% of patients with COVID‐19 had evidence of pulmonary fibrotic sequelae persisting at 1‐year follow‐up. Fibrosis is promoted by profibrotic factors, primarily transforming growth factor‐β (TGF‐β), with intense profibrotic processes promoting epithelial‐to‐mesenchymal and endothelial‐to‐mesenchymal transition.^(^ ^99^ ^)^ In most patients, TGF‐β secretion from the affected lung is able to promote repair and resolution of infection‐induced damage, but in patients with severe COVID‐19 SARS‐2 infection can lead to excessive TGF‐β signaling and fibrosis.^(^ ^100^ ^)^ 1,25(OH)2D could markedly inhibit the activation of TGF‐β signaling pathways, downregulate the upregulation of fibronectin and collagen expression, and consequently inhibit the transdifferentiation of TGF‐β1 and stimulated lung epithelial cells into myofibroblasts.^(^ ^101^ ^)^

On the other hand, activation of the renin‐angiotensin system (RAS) induces pulmonary fibrosis in both transgenic animals and disease models^(^ ^102^ ^)^ and is recognized as an important pathogenic factor in the pathogenesis of pulmonary fibrosis.^(^ ^103^ ^)^ VDES has an anti‐fibrotic role by negatively regulating RAS. Vitamin D deficiency has been shown to activate RAS and knockout mice for VDR‐caused renin overexpression, resulting in more angiotensinogen transformed into angiotensin II. Importantly, Ang II can activate SMAD2/3 directly by the ERK/p38 pathway and regulate TGF‐β/SMAD3 signaling at multiple levels.^(^ ^104^ ^)^

Acute respiratory distress syndrome is the common immunopathological event that SARS‐CoV‐2 infection shares with SARS‐CoV and MERS‐CoV.^(^ ^105^ ^)^ There is currently no Food and Drug Administration (FDA)‐approved drug treatment that is effective for ARDS, and treatment remains supportive with lung‐protective mechanical ventilation.^(^ ^106^, ^107^ ^)^ Identifying early effective and safe therapeutic strategies remains a challenge. Therefore, stimulation of the VDR to reduce ARDS in patients with SARS‐CoV‐2 coronavirus could be a therapeutic opportunity.^(^ ^55^ ^)^

#### Clinical data

##### Observational studies

A few studies evaluated the risk of infection with SARS‐CoV‐2 and the vitamin D status before the disease. Several studies found or suggested an increased susceptibility when serum 25OHD falls below 20 ng/mL,^108^, ^109^, ^110^, ^111^, ^112^ ^)^ but other studies did not confirm this observation.^(113^ ^)^ A systematic review and meta‐analysis concluded that serum 25OHD was about 5 ng/mL lower in COVID‐19‐infected patients compared with those not infected. However, the timing of blood drawing in the controls were not simultaneously with the infected patients, and overall, most of these observational studies were of poor quality.^(^ ^114^ ^)^

A large number of studies evaluated the severity of the course of COVID‐19 infections and the vitamin D status before the infection or at the time of diagnosis or hospitalization. Two recent reviews extensively analyzed these results^(^ ^114^, ^115^ ^)^ based on 31 observational studies^(^ ^114^ ^)^ or 17 studies^(^ ^115^ ^)^ and came to quite different conclusions. Bassatne and colleagues^(114^ ^)^ concluded that the association of serum 25OHD below 20 ng/mL and COVID‐19 outcomes was rather uncertain. In this meta‐analysis including 13 cross‐sectional and 7 cohort studies, there was a trend to higher mortality (odds ratio [OR] = 2.1; 95% confidence interval [CI] 0.9–4.8) in subjects with the lowest 25OHD concentrations. A similar (non‐significant) trend was also found for need of ICU admission, mechanical ventilation, or length of hospital stay.^(^ ^114^ ^)^ The overall analysis of the reported studies was problematic because of the high degree of heterogeneity and different definitions of vitamin D status and outcomes. The meta‐analysis of Wang and colleagues^(^ ^115^ ^)^ restricted the analysis to 17 studies on 2756 patients with proven COVID‐19 infections. Vitamin D deficiency was associated with significantly higher mortality (OR = 2.47; 95% CI 1.50–4.05), higher rates of hospital admissions (OR = 2.18; 95% CI 1.48 to 3.21) and longer hospital stay (0.52 days; 95% CI 0.25–0.80) compared with subjects with a normal vitamin D status. Admission to ICU was not significantly different. Based on all available studies, it is likely that a poor vitamin D status is one of several risk factors for the severity of COVID‐19 infections.

**Fig. 4:**
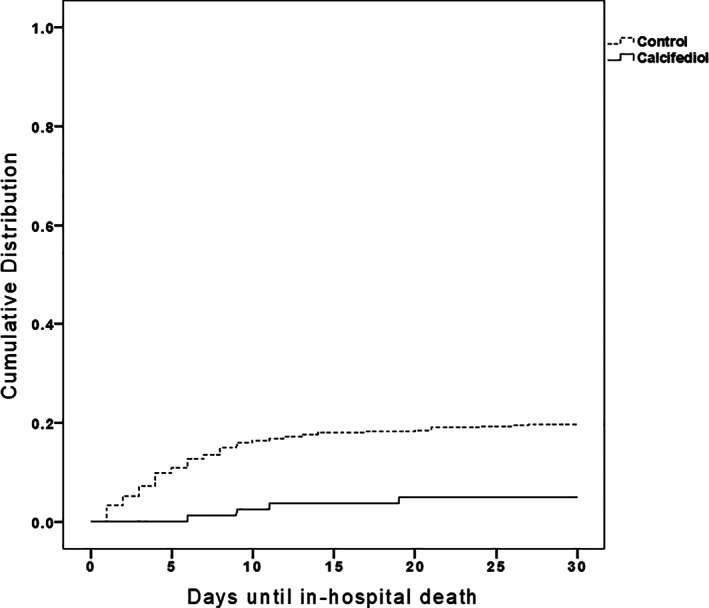
Retrospective, multicenter cohort study of calcifediol treatment and outcome of COVID‐19 infection.^(^ ^116^ ^)^ Patients (*n* = 537) hospitalized because of COVID‐19 infections received the best available treatment for SARS‐CoV‐2 infection and standard care for preexisting comorbidities. Treatment groups based on having received from admission: (1) oral calcifediol (25OH D3) in soft gelatin capsules (0.532 mg), then oral calcifediol (0.266 mg) on days 3 and 7, and then weekly until discharge or ICU admission (*n* = 79); (2) no treatment with calcifediol (*n* = 458). Cumulative distribution of patients presenting in‐hospital death according to treatment groups. Patients hospitalized with COVID‐19, treatment with calcifediol, compared with those not receiving calcifediol showed a significantly lower in‐hospital mortality during the first 30 days (5% versus 20%, *p* < 0.01).

In a retrospective study of patients hospitalized for laboratory‐confirmed COVID‐19 infection, patients from five hospitals in southern Spain received or not calcifediol (similar schedule as in the pilot study mentioned below). Patients from one hospital received the option to receive calcifediol, whereas this option was not available in the other hospitals. General treatment was otherwise similar. Slightly more patients in the treatment group had one or more comorbidities at baseline. In‐hospital mortality during the first 30 days was 17.5%. The OR of death for patients receiving calcifediol (mortality rate of 5%) was 0.22 (95% CI 0.08–0.61) compared with patients not receiving such treatment (mortality rate of 20%; *p* = 0.0005). In the multivariable logistic regression model, there were significant differences in mortality for patients receiving calcifediol compared with patients not receiving (OR = 0.16; 95% CI 0.03–0.80)^(^ ^116^ ^)^ (Fig. 4). {FIG4}

Another observational cohort study included patients admitted to COVID‐19 wards of Hospital del Mar, Barcelona, Spain. Patients previously treated with calcifediol were excluded. Of 838 patients, 447 received calcifediol (532 μg on day 1 plus 266 μg on days 3, 7, 15, and 30), whereas 391 were not treated at the time of hospital admission. The prescription of calcifediol was based on the ward they were assigned to, based on availability of beds. In five of eight wards, patients received calcifediol, whereas this was not the case in the other three wards. Treatment was otherwise similar and there were no significant baseline differences in patient characteristics. ICU assistance was required by 102 (12.2%) participants. Of 447 patients treated with calcifediol at admission, 20 (4.5%) required ICU compared with 82 (21%) of 391 nontreated (*p* < 0.001). Logistic regression of calcifediol treatment on ICU admission, adjusted by age, sex, linearized 25OHD levels at baseline, and comorbidities, showed that treated patients had a reduced risk to require ICU (OR = 0.13; 95% CI 0.07–0.23). Overall mortality was 10%. In the intention‐to‐treat analysis, 21 (4.7%) of 447 patients treated with calcifediol at admission died compared with 62 patients (15.9%) of 391 nontreated (*p* = 0.0001). Adjusted results showed a reduced mortality risk with an OR = 0.21 (95% CI 0.10–0.43). Calcifediol treatment at hospital admission thus significantly reduced the need for ICU support and reduced mortality.^(^ ^117^ ^)^

Very recently, data from three retrospective cohort studies have been reported.^(^ ^118^, ^119^, ^120^ ^)^ One cohort of 15,968 patients, including all hospitalized patients with a confirmed diagnosis of COVID‐19 between January and November 2020 in Andalusia (Spain), one of the largest regions in Europe with the size of an average country, found 570 patients prescribed cholecalciferol, and 374 calcifediol, 15 days before hospitalization. When they extended the period to 30 days before hospitalization, the figures increase to 802 and 439, respectively. The Kaplan–Meier survival curves and hazard ratios assessed consistently support an association between prescription of these VDES metabolites and patient survival. This association was stronger for calcifediol (hazard ratio [HR] = 0.67; 95% CI 0.50–0.91) than for cholecalciferol (HR = 0.75; 95% CI 0.61–0.91), when prescribed 15 days between hospitalization. This protective effect decreases when a longer period since prescription (30 days) is considered (HR of calcifediol = 0.73; 95% CI 0.61–0.91) and cholecalciferol (HR = 0.88; 95% CI 0.75–1.03), suggesting that the closer the treatment is to hospitalization, the stronger the association and the stronger the protective effect.^(^ ^118^ ^)^ A retrospective cohort study in the Barcelona area (Catalonia; Spain) assessed the risk of COVID‐19 infection in subjects who had been prescribed vitamin D or calcifediol during the previous 4 months^(^ ^119^ ^)^ according to data generated from the local public health system. The hazard ratio of infection was slightly, but significantly, lower in subjects taking cholecalciferol (HR = 0.95; 95% CI 0.91–0.98) but not in subjects taking calcifediol. In a small subset of the population, serum 25OHD levels had been measured. When evaluating COVID‐19 results in patients with serum 25OHD levels >30 ng/mL, supplemented with cholecalciferol, they reported that the rate of SARS‐CoV‐2 infection (HR = 0.66; 95% CI 0.57–0. 77), the risk of severe COVID‐19 (HR = 0.72; 95% CI 0.52–1.00), and COVID‐19 mortality (HR = 0.66; 95% CI 0.46–0.93) were significantly lower compared with vitamin D‐deficient patients (25OHD <20 ng/mL) who were not receiving supplementation. Similarly, when the treatment given was calcifediol, both the rate of SARS‐CoV‐2 infection (HR = 0.69; 95% CI 0.61–0.79), the risk of severe COVID‐19 (HR = 0.61; 95% CI 0.46–0.81), and notably COVID‐19 mortality (HR = 0.56; 95% CI 0.42–0.76) were significantly lower.^(^ ^120^ ^)^

The same research group, in a retrospective cohort study throughout Catalonia in patients with advanced chronic kidney disease (stage 4 and/or 5), evaluated the effect of calcitriol, the hormonal form of VDES.^(^ ^120^ ^)^ After propensity score matching, 6252 calcitriol patients and 12,504 matched control patients were included in the study. Prescribing calcitriol to patients was associated with a lower risk of SARS‐CoV‐2 infection (HR = 0.78; 95% CI 0.64–0.94), lower risk of severe COVID‐19, and lower COVID‐19 mortality (HR = 0.57; 95% 0.41–0.80). In treated subjects, regardless of renal function, there was an inverse association between mean daily dose of calcitriol and severity of COVID‐19 or mortality.

##### Intervention studies

The NIH trial register mentions a very large number of studies dealing with vitamin D supplementation and infection or severity of COVID‐19. However, so far, few randomized controlled trials (RCTs) have published real results. A Brazilian RCT evaluated the effect of a single high dose of vitamin D (200,000 IU) on COVID‐19 outcome of 240 patients with a mean baseline serum 25OHD of about 20 ng/mL. No effects were observed on length of hospital stay, mechanical ventilation, ICU admission, or mortality.^(^ ^121^ ^)^

In a parallel, open‐label, randomized, double‐masked pilot clinical trial conducted at the Hospital Universitario Reina Sofia in Cordoba, Spain, in 76 consecutive patients hospitalized for COVID‐19, treated with oral calcifediol (0.532 mg on the day of admission, then 0.266 mg on days 3 and 7, and then weekly until discharge, death, or ICU admission), calcifediol treatment significantly reduced the need for ICU admission (OR = 0.03; 95% CI 0.003–0.25).^(^ ^122^ ^)^ In follow‐up of this pilot trial, several additional studies have explored whether calcifediol might improve COVID‐19 outcomes.

## Discussion

SARS‐CoV‐2 is a novel coronavirus that rapidly spread around the world as a serious infection at a rate not observed in the last century.^(^ ^123^ ^)^ Its spread has had a devastating impact on health systems and national economies around the world. Current management strategies for SARS‐2/COVID‐19 range from the implementation of preventive (social) measures and vaccination to control viral shedding to the implementation of supportive care when indicated and the use of (repurposed) drugs. There are no effective antiviral drugs to prevent or markedly mitigate the disease. Because de novo development of specific anti‐SARS‐CoV‐2 drugs and vaccines takes time, clinical research has focused on drug repurposing.^(^ ^124^, ^125^ ^)^ The susceptibility for the disease is greater in subjects with predisposing factors such as age, male sex, obesity, and diabetes, chronic lung diseases, and cardiovascular problems.^(^ ^126^ ^)^ Most of these risk factors are known to be associated with a poor vitamin D status.^(^ ^127^ ^)^ Therefore, since the beginning of the pandemic, vitamin D deficiency was suspected as a possible contributing factor either to disease susceptibility or to a more severe course of the disease.

Currently, we have evidence of a deregulated vitamin D endocrine system in lung and immune cells of SARS‐CoV‐2‐infected patients.^(^ ^128^ ^)^ Moreover, a recent study on systematic drug repurposing for COVID‐19 based on machine learning has found that, among others, the VDR protein could have a protector effect over pathways affected by the SARS‐CoV‐2 infection,^(^ ^129^ ^)^ suggesting a potential protecting role for VDES metabolites such as cholecalciferol, calcifediol, or calcitriol. This hypothesis is plausible as the vitamin D endocrine system is known to regulate a large number of genes or cells involved in the immune system.^(^ ^4^ ^)^ In addition, the major target of the SARS‐2 virus, the lung, has recently been identified as a target of the VDR system.^(^ ^128^ ^)^ However, as always, association or plausible hypotheses do not prove causality. In the field of vitamin D, many strong associations and plausible mechanism have linked a poor vitamin D status with a large number of diseases,^(^ ^1^ ^)^ but an increasing number of supplementation or Mendelian randomization studies cast doubt on a major causal role of vitamin D in such extraskeletal diseases.^(^ ^130^ ^)^

There are many plausible mechanisms that suggest that 25OHD deficiency may be a risk factor for the susceptibility for COVID‐19, in line with the meta‐analysis confirming a modest beneficial effect of vitamin D supplementation on upper respiratory infections.^(^ ^53^ ^)^ However, if so, it is only a modest risk factor in comparison with other major risks factors. Because there are no RCTs dealing with the risk of COVID‐19 infections with or without vitamin D supplementation, there is obviously also no clear threshold below which subjects are more prone to that infection. From data dealing with upper respiratory infections, vitamin D deficiency (25OHD below 20 ng/mL) conveys a small increased risk and supplementation with modest doses (usually below 1000 IU/D) generated a small protective effect.^(^ ^53^, ^54^ ^)^ It is more plausible that a good vitamin D status may have beneficial effects on the course or consequences of COVID‐19 infections. This is mainly based on the effects of 1,25(OH)2D on tapering down excessive inflammation leading to acute respiratory distress syndrome and other mechanisms protecting the lung epithelium from viral or inflammatory damage. The observational data are not uniformly in line with this conclusion. One meta‐analysis concluded that there was a non‐significant trend for an association between a poor vitamin D status and several health outcomes of COVID‐19‐infected patients. Another meta‐analysis, however, based on observational studies dealing with PCR‐proven cases and contemporary controls, suggested a modest link between better vitamin D status and major outcomes of the infection with regard to requirements for ICU admission or mortality.^(^ ^114^, ^115^ ^)^

25OHD deficiency may not be the most dominant factor in comparison with other risks such as age, obesity, hypertension, chronic lung diseases, diabetes, or cardiovascular diseases, but it is certainly an easily modifiable factor that deserves more widespread attention.

Unfortunately, few randomized trials are yet available. One study did not show beneficial effects of a large bolus dose on the outcome of COVID‐19. One pilot study using calcifediol, however, revealed a major beneficial effect on requirement of ICU admission. These positive effects of calcifediol are further reinforced by observational data in three other recent Spanish trials. These studies were not designed to define which 25OHD target level generated the highest protection. However, the dose of calcifediol used as loading dose is expected to rapidly increase mean serum 25OHD above 40 ng/mL. None of the studies included a dose‐response question. Fortunately, a large number of studies are ongoing (as reported in the NIH trial database) as to demonstrate whether improvements of the vitamin D status may favorably affect the struggle against the COVID‐19 pandemic.

Vaccination is evidently the best strategy to defeat the COVID‐19 pandemic. There has been some speculation (without evidence from trials) that a poor vitamin D status may decrease the immune response to such vaccination. A meta‐analysis, however, found no link between vitamin D status and immune response to influenza vaccination.^(^ ^131^ ^)^

Observational data strongly suggest that supplementation with vitamin D or, especially, calcifediol may decrease the severity of this disease as demonstrated by reduced need for intensive care and decreased mortality risks. Further large, randomized trials are needed. In the meantime, we recommend rapid correction of 25OHD deficiency in all subjects possibly exposed to this coronavirus. This cost‐effective and widely available treatment could have positive implications for the management of COVID‐19 worldwide.

## Conflict of Interest

RB received small lecture fees from Abiogen (Italy), FAES‐Farma (Spain), and Fresenius (Germany). JMQG received small lecture fees from Amgen (Spain) and FAES‐Farma (Spain).

### Peer Review

The peer review history for this article is available at https://publons.com/publon/10.1002/jbm4.10576.
